# Impact of sleep disorders on behavioral issues in preschoolers with autism spectrum disorder

**DOI:** 10.3389/fpsyt.2023.1181466

**Published:** 2023-04-26

**Authors:** Giacomo Distefano, Sara Calderoni, Fabio Apicella, Angela Cosenza, Roberta Igliozzi, Giuseppina Palermo, Raffaella Tancredi, Giovanna Tritto, Francesco Craig, Filippo Muratori, Marco Turi

**Affiliations:** ^1^Department of Clinical and Experimental Medicine, University of Pisa, Pisa, Italy; ^2^IRCCS Fondazione Stella Maris, Pisa, Italy; ^3^Stella Maris Mediterraneo Foundation, Chiaromonte, Italy; ^4^Department of Cultures, Education and Society, University of Calabria, Cosenza, Italy; ^5^Department of Human and Social Studies, University of Salento, Lecce, Italy

**Keywords:** sleep disorders, autism spectrum disorders, preschoolers, psychiatric comorbidities, repetitive behaviors, behavioral problems

## Abstract

**Background:**

Sleep disorders are one of the most common problems in children with Autism Spectrum Disorder (ASD). However, they often tend to be underdiagnosed and incorrectly treated in clinical practice. This study aims to identify sleep disorders in preschool children with ASD and to explore their relationship with the core symptoms of autism, the child’s developmental and cognitive level as well as the psychiatric comorbidities.

**Methods:**

We recruited 163 preschool children with a diagnosis of ASD. The Children’s Sleep Habits Questionnaire (CSHQ) assessed sleep conditions. Multiple standardized tests were used to evaluate intellectual abilities, the presence of repetitive behaviors (through the Repetitive Behavior Scale-Revised), as well as the emotional-behavioral problems and the psychiatric comorbidities (through the Child Behavior Checklist -CBCL 1^1/2^-5).

**Results:**

The results showed that poor disorders had consistently higher scores in all areas assessed by the CSHQ and on the CBCL across all domains. The correlational analysis showed that severe sleep disorders were associated with higher scores in internalizing, externalizing, and total problems at the CBCL syndromic scales, and in all DSM-oriented CBCL subscales. Moreover, we found that the association between sleep disorders and restricted and repetitive behaviors (RRBs) is explained by the anxiety-related symptoms.

**Conclusion:**

Based on these findings, the study recommends that screening for sleep problems followed by early intervention should constitute a routine part of clinical practice for children with ASD.

## Introduction

Autism spectrum disorders (ASD) refer to a diverse set of neurodevelopmental conditions that are marked by noticeable deficits in social interaction and communication, as well as unusual sensori-motor behaviors or interests that may be repetitive or restrictive in nature. The symptoms caused by ASD appear from early childhood and negatively impact the child’s daily functioning (1).

Sleep disorders (SD) are certainly among the most reported problems in individuals with ASD of all ages and have a negative impact on daily functioning, learning, and behavior, not only of the person with ASD but also of the entire family (2). Indeed, an up-to-date review that used the Diagnostic and Statistical Manual of Mental Disorders Fifth Edition (DSM-5)'s most stringent criteria to identify sleep disorders emphasized that the ASD population had a prevalence rate of 13%, which is significantly higher than the 3.7% rate observed in the general population (3). Studies specifically focusing on pediatric samples report that one-half up to two-thirds of children with ASD might have SD (4–7) and underline the importance of early diagnosis and treatment to avoid the tendency of these disorders to become chronic (6, 8). A different comprehensive assessment of sleep disturbances using both subjective and objective measures discovered notable sleep difficulties in children with ASD in comparison to their typically developing counterparts. These issues included significantly reduced total sleep duration, prolonged time taken to fall asleep, and lower quality of sleep (9).

While there is substantial literature on sleep disorders in individuals with ASD, only a few studies have specifically focused on the preschool population. A study by Krakowiak and colleagues investigated the sleep patterns of children between the ages of 2 and 5 with ASD, as compared to children with other types of developmental delays (DD) and typical development (TD). The authors used the Children’s Sleep Habits Questionnaire (CSHQ), which was administered to parents to collect data on their children’s sleep patterns. They observed that at least one sleep issue occurred frequently in 53% of children with ASD, which is higher than the 46% observed in children with DD and the 32% noted in TD peers. The most common sleep difficulties reported in the ASD group were trouble falling asleep and waking up during the night (10). In a similar vein, a more recent study using a larger cohort of patients aged 2 to 5 years found similar results using the CSHQ, comparing different groups (ASD, DD with ASD symptoms, DD without ASD, and TD). Caregiver reports of sleep issues in children with ASD were 47%, DD with ASD were 57%, DD without ASD were 29%, and TD were 25% (11). Both studies emphasized the significance of early detection of sleep issues due to the effects of inadequate sleep on a child’s behavior throughout the day and on their quality of life (10, 11).

Sleep questionnaires are one of the most commonly used tools to investigate subjective sleep characteristics and problems in children. These questionnaires are also important in highlighting the impact of sleep problems on the quality of life of families with children who have ASD and SD. One of the most frequently used questionnaires is the Children’s Sleep Habits Questionnaire (CSHQ), which was designed specifically for the evaluation of children by Owens et al. in 2000 (12). This questionnaire was later validated in toddlers and preschool children with TD, ASD, and other DD by Goodlin-Jones et al. (13). Eventually, it was specifically modified for the ASD population by Katz et al. (14). The validity and usefulness of the CSHQ in the ASD population and in particular in preschool children have been confirmed by several studies (9, 15, 16).

Other similar questionnaires, such as the Sleep Disturbance Scale for Children (SDSC), are used to evaluate sleep disturbances in infants and toddlers (17). The SDSC was recently used by Romeo et al. to assess sleep disorders in preschoolers with ASD, and revealed that 46% of children with ASD have at least one SD, compared to 15% in the control group. Difficulty in initiating and maintaining sleep, excessive daily somnolence, and sleep hyperhidrosis showed higher subscale scores (18).

Overall, screening for sleep problems in all children with ASD should be recommended, and a sleep specialist should be consulted when comorbid sleep disorders are suspected (19). Parental questionnaires have the benefit of saving time and costs, can be used as a follow-up instrument, and can measure a broad range of sleep parameters.

Recent literature reviews have analyzed the relationship between sleep disturbance and daytime behavior in the ASD population (4, 20–22). A large cross-sectional study on 1,193 children with ASD showed that children with ASD and SD demonstrated higher internalizing and externalizing behavior problems identified by the Child Behavior Check-List (CBCL) (23). Malow et al. investigated the correlation between parentally reported sleep problems in ASD, objective polysomnographic findings, and measures of daytime behavior. The authors divided ASD children into two groups: poor sleepers and good sleepers, based on the CSHQ total score, and observed higher T-scores on all scales of the CBCL in the poor sleeper group, with more clinical scores in affective, attention, and anxious/depressed subscales (24).

The vast majority of studies investigating the correlation between SD and daytime behavior in ASD are conducted with school-aged samples, while only a few have focused on the pre-school population. Among these, a recent study using the CSHQ and the CBCL found a remarkable difference in the effect of parasomnias on internalizing, externalizing, and total problems in children with ASD (25).

In the current study, we focus on an ASD population of infants and toddlers, with an age range between 21 and 66 months. Sleep problems in this age range are certainly less investigated in literature compared to older ages. We used the CSHQ filled in by parents to identify good and poor sleepers in our sample of children with ASD, with the aim of (a) assessing the prevalence of SD in our population sample, and (b) identifying possible correlations between sleep problems and autistic core symptoms, cognitive functioning, restricted and repetitive behaviors, as well as emotional-behavioral problems through standardized tools and questionnaires used in daily clinical practice.

## Participants

Between January 2020 and August 2022, we conducted a cross-sectional study (refer to Table 1) on 163 preschoolers with a diagnosis of ASD. These children, aged between 21 and 66 months (with a mean age of 43.37 months and SD of 12.56 months) were recruited from two different Italian child care centers. Of the 163 children, 127 were boys and 36 were girls, with 50 children from IRCCS Fondazione Stella Maris in Pisa and 113 children from Stella Maris Mediterraneo Foundation in Matera. In the current study, we enrolled participants who had received a diagnosis of ASD in accordance with DSM-5 criteria (1). A multidisciplinary team, consisting of a senior child psychiatrist and an experienced research child psychologist, was responsible for performing the diagnosis. To confirm the clinical diagnosis, we used the Autism Diagnostic Observation Schedule, Second Edition (ADOS-2) (26), which is considered the gold-standard semi-structured instrument for observing and assessing communication abilities, social interaction, play quality, and imagination in children.

**Table 1:** Demographic, clinical characteristics in the total sample (*n* = 163), and in each sleep group.

	Whole sample (*n* = 163)	Good sleeper (*n* = 89)	Poor sleeper (*n* = 74)	*t*-test or *X*^2^	Value of *p*	Effect size
Age (months)						
*M* (SD)	43.37 (12.56)	43.56 (13.1)	43.14 (11.96)	*t*_(161)_ = 0.21	*p* = 0.83	–
Range	21–66	25–66	21–66			
*Gender (F:M)*	36 (22%): 127 (78%)	19 (22%): 70 (78%)	17 (23%): 57 (77%)	*X^2^ = 0.80*	*p* = 0.85	*–*
Performance IQ						
*M* (SD)	65.59 (22.90)	66.94 (22.04)	63.96 (23.94)	*t*_(161)_ = 0.82	*p* = 0.40	*–*
Range	13–133	23–122	13–133			
Social affect ADOS						
*M* (SD) range	14.03 (4) 4–20	13.88 (3.82) 4–20	14.20 (4.22) 4–20	*t*_(161)_ = −0.48	*p* = 0.91	*–*
RRB ADOS						
*M* (SD) range	3.76 (1.89) 0–8	3.85 (1.93) 0–8	3.65 (1.86) 0–8	*t*_(161)_ = 0.66	*p* = 0.62	*–*
ADOS total score						
*M* (SD) range	17.80 (4.88) 5–28	17.76 (4.65) 7–28	17.85 (5.18) 5–27	*t*_(161)_ = −0.14	*p* = 0.51	*–*

Significant comparisons are highlighted in bold (*p* < 0.05).

We excluded any cases of syndromic autism or recognized causes of ASD, as well as children who had used psychotropic medications in the 2 months before the evaluation. All participants were residents of Italy. We conducted the study in accordance with the ethical standards for good practice and the guidelines outlined in the Declaration of Helsinki. Written informed consent was acquired from the parent or caregiver of each participant.

## Cognitive assessment

Given the variations in verbal skills and functioning levels in children with ASD, a variety of standardized tests have been used to measure their intellectual abilities. These tests include the Leiter International Performance Scale-Revised (LIPS-R) (27), the Griffiths Mental Developmental Scales-Extended-Revised (GMDS-ER) (28), and the Italian version of the Wechsler Preschool and Primary Scale of Intelligence (WPPSI) (29).

We selected these tests to ensure a comprehensive assessment of intellectual abilities in children with ASD, considering the diverse range of skills and abilities that may be present in this population. In cases where a mental age (MA) was provided by the test, we estimated the child’s IQ by dividing their MA by their chronological age (CA) and multiplying the result by 100: MA/CA × 100. For this study, we focused on non-verbal IQ scores (performance IQ or pIQ).

## Children’s Sleep Habits Questionnaire

The Children’s Sleep Habits Questionnaire (CSHQ) is a widely accepted and scientifically validated questionnaire used to assess sleep-related problems in children aged 3 to 10 years old (12). It has been used with even younger children, as young as 2 years old (13), and has also been applied in studies with autistic adolescents (30). The Children’s Sleep Habits Questionnaire (CSHQ) covers various aspects of sleep, including sleep onset, sleep maintenance, parasomnias, and daytime sleepiness. This questionnaire is widely used in research to evaluate the prevalence and severity of sleep disturbances in children and to assess the effectiveness of interventions aimed at improving sleep. In clinical settings, the CSHQ is used to identify children with sleep problems and track changes in their sleep patterns over time. The CSHQ provides both a total score and subscale scores across different sleep problems. A total score of 41 is suggested to be the cut-off for identifying sleep problems (12). The psychometric properties of the CSHQ are provided alongside reference values for subscales, which are useful for comparing clinical and community groups (12). In the present investigation, based on the approach of previous studies (11, 23), CSHQ total scores of 48 and above were defined as a more conservative cut-off to identify children having SD. The reason for using a cut-off of 48 was first of all to avoid an overestimation of sleep disturbances in children of such a young age as in our sample, who are very often subject to sleep disturbances that are not necessarily pathological and because the CSQH is calibrated on a larger reference population in terms of age (12). This methodological choice is also supported by literature: indeed, there are several different works that have highlighted how the cut-off of 48 had a higher sensitivity in different populations both with TD (31, 32), and with neurodevelopmental disorders, such as DD and young children with ASD in very large samples (11, 23).

Based on the clinical cut-off of the CSHQ, we divided the whole sample into good sleepers (CSQH Total Score < 48) and poor sleepers (CSQH Total Score ≥ 48), as shown in Table 1.

## Child Behavior Checklist 1½–5

The Child Behavior Checklist (CBCL 1½–5) (33), which is widely used to evaluate children’s behavior, was used in this study. The questionnaire involves parents rating a child’s behaviors using a three-point scale, with 0 meaning “not true,” 1 meaning “somewhat or sometimes true,” and 2 meaning “very true or often true.” The CBCL generates scores for seven syndrome scales, three summary scales, and five DSM-Oriented scales (DOS). Clinical significance is defined as a T-score of 64 or higher for summary scales and a T-score of 70 or higher for syndrome and DOS. A borderline clinical range is indicated by values between 60 and 63 for summary scales or between 65 and 69 for syndrome and DOS. Clinically relevant values are those above 65 for the other scales and below 60 for the summary scales. According to other research on screening (34–36) and psychiatric comorbidity (37–39) in young children with ASD, we adopted the borderline cut-off score (T score 60 for summary scales and T score 65 for DOS) in this investigation. Recently, it has been suggested that the adoption of lower clinical CBCL thresholds for preschoolers is being considered, as using identical thresholds for both preschoolers and school-age children is being questioned (40). Moreover, research has indicated that parents and teachers evaluate this age group of children using less strict standards than they do older children, both in terms of internalizing (41), and externalizing behaviors (42).

## Repetitive Behavior Scale-Revised

The Italian version of the Repetitive Behavior Scale-Revised (RBS-R) (43, 44) was used to measure repetitive behaviors in people with ASD. The RBS-R is a 43-item questionnaire completed by caregivers, which assesses a wide range of restricted and repetitive behaviors observed in the individual over the past month. It is divided into six subcategories that cover different types of behaviors, such as compulsive, self-injurious, ritualistic, restricted, insistence on sameness, and stereotypical behaviors. Studies on the RBS-R have found that it can be divided into either five or three factors (45, 46). The questionnaire produces both a count score and a severity score, with the count score reflecting the number of items endorsed by the parents or caregivers and the severity score reflecting the parents or caregivers’ assessment of the severity of the behavior. Previous studies have recommended using the count score in analysis to reduce bias and increase accuracy (47).

## Data analysis

We examined the normality of continuous variables through both skewness tests and Kolgomorov-Smirnov testing. For categorical and continuous independent variables, we utilized descriptive analysis, chi-square analysis, and t-tests, respectively. To assess whether there were differences in age, PIQ, CSHQ scores, and CBCL scales across all groups, we performed an independent sample t-test. We conducted both simple and partial correlation analyses to investigate the relationships between sleep and clinical variables, and used logistic regression to examine any associations between sleep and behavioral problems. Appropriate effect size was evaluated in accordance with the statistical method chosen for the analysis (e.g., Cohen’s *d* for independent sample *t*-tests).

## Results

In our study, we recruited a sample of 163 children and used a cut-off point from the CHSQ to divide them into two groups: those who were considered to be good sleepers -GS- (CHSQ total score < 48), referred to as children without a clinical total score on the CHSQ, and those who were considered to be poor sleepers -PS- (CHSQ total score ≥ 48), referred to as children with a clinical total score on the CHSQ. After applying this cut-off, it was found that 54% of the sample, or 89 children, were classified as GS, and 46% of the sample, or 74 children, were classified as PS. The mean CSHQ subscale scores for the whole sample were compared with the standard values for a community group (12), showing that ASD children had clinically higher scores on the following CSHQ subscales: bedtime resistance (54%), sleep onset delay (23%), sleep duration (16%), sleep anxiety (26%), night wakings (25%), parasomnias (11%), sleep disordered breathing (6%), daytime sleepiness (1%).

Table 1 demonstrates that both groups were similar in terms of age and PIQ (age: value of *p* = 0.083: PIQ: value of *p* = 0.40), and there were no statistically significant differences in the male to female ratio between the groups (value of *p* = 0.85). Additionally, we found that there were no statistically significant differences in autism symptoms between the sleep groups as evaluated by the ADOS-2, which includes the ADOS Total Score, Social Affect (SA), and Repetitive Restricted Behaviors (RRB) (ADOS Total Score: value of *p* = 0.51; SA: value of *p* = 0.91; RRB: value of *p* = 0.62). Table 2 compares the CSHQ scores among the two sleep groups. As expected, the group with poor sleep had consistently higher scores in all areas assessed by the CSHQ (all value of *p* < 0.05), indicating an overall worsening of all aspects of sleep investigated by the CSHQ.

**Table 2:** Differences in CSHQ scales between poor and good sleepers.

	Good sleeper (*n* = 89)	Poor sleeper (*n* = 74)	*t*-test	Value of *p*	Effect size
Bedtime resistance *M* (SD)	9.33 (2.04)	12.75 (1.98)	*t*_(161)_ = −10.60	***p* < 0.0001**	*d* = 1.66
Sleep onset delay *M* (SD)	1.53 (0.67)	1.98 (0.86)	*t*_(161)_ = −4.05	***p* = 0.001**	*d* = 0.73
Sleep duration *M* (SD)	3.40 (0.90)	4.49 (1.57)	*t*_(161)_ = −5.84	***p* < 0.0001**	*d* = 0.89
Sleep anxiety *M* (SD)	5.33 (1.13)	7.80 (1.67)	*t*_(161)_ = −9.91	***p* < 0.0001**	*d* = 1.65
Night wakings *M* (SD)	4.13 (1.16)	5.21 (1.48)	*t*_(161)_ = −5.57	***p* < 0.0001**	*d* = 0.73
Parasomnias *M* (SD)	7.69 (0.91)	9.36 (1.87)	*t*_(161)_ = −7.43	***p* < 0.0001**	*d* = 1.13
Sleep disorder breathing *M* (SD)	3.13 (0.65)	3.46 (0.92)	*t*_(161)_ = −2.98	***p* = 0.003**	*d* = 0.47
Daytime sleepiness *M* (SD)	7.84 (1.57)	10.54 (2.61)	*t*_(161)_ = −7.57	***p* < 0.0001**	*d* = 1.17
Total score M(SD)	42.46 (3.25)	55.64 (6.16)	*t*_(161)_ = −16.94	***p* < 0.0001**	*d* = 2.59

Significant comparisons are highlighted in bold (*p* < 0.05).

Regarding the association between emotional-behavioral problems and type of sleeper, Table 3 shows the distribution of the CBCL scores on the summary scales (Total, Internalizing and Externalizing problems), and on DOS scales among the two sleep groups. Simple group comparison on the CBCL scales showed higher T-scores on all domains for the PS when compared with GS (all value of *p*s <0.05).

**Table 3:** Differences in DOS and Summary Scale of CBCL between poor and good sleepers.

	Good sleeper (*n* = 89)	Poor sleeper (*n* = 74)	*t*-test	Value of *p*	Effect size
Internalizing problems					
*M* (SD)	56.16 (10.11)	65.38 (8.82)	*t*_(161)_ = −5.07	***p* < 0.0001**	*d* = 0.79
Externalizing problems					
*M* (SD)	53.75 (9.49)	61.05 (10.05)	*t*_(161)_ = −3.89	***p* < 0.0001**	*d* = 0.90
Total problems					
*M* (SD)	55.13 (9.87)	65.42 (10.25)	*t*_(161)_ = −5.26	***p* < 0.0001**	*d* = 0.82
*Affective problems*					
*M* (SD)	56.28 (7.68)	65.71 (9.20)			
Number case	11 (12.4)	35 (47.3)	*t*_(161)_ = −7.22	***p* < 0.0001**	*d* = 1.12
Anxiety problems					
*M* (SD)	53.66 (5.08)	61.44 (8.86)	*t*_(161)_ = −5.92	***p* < 0.0001**	*d* = 0.91
Number case	4 (4.5)	23 (31.1)			
PDP					
*M* (SD)	66.68 (8.63)	73.71 (9.52)	*t*_(161)_ = −3.35	***p* = 0.001**	*d* = 0.52
ADHD					
*M* (SD)	57.69 (6.88)	61.96 (7.62)	*t*_(161)_ = 3.47	***p* = 0.001**	*d* = 0.46
Number case	24 (27)	34 (45.9)			
Oppositional problems					
*M* (SD)	54.37 (5.88)	58.44 (7.93)	*t*_(161)_ = −3.68	***p* = 0.001**	*d* = 0.57
Number case	11 (12.5)	22 (29.7)			

Significant comparisons are highlighted in bold (*p* < 0.05). N Case [Percent of Case (%) above borderline cut-off for CBCL DOS Scales].

Considering the whole sample, a bivariate Pearson correlation also revealed a statistically significant association between the occurrence of sleep disorders (CSHQ Total Score) and the presence of psychiatric comorbidities, i.e., higher sleep problems were associated with higher T-Score on DOS scales (Table 4). These results are consistent also controlling for age, gender and PIQ (all value of *p*<0.05).

**Table 4:** Pearson correlations among the CSHQ total score and the CBCL DOS Scales.

	CSHQ-total score	Affective problems	Anxiety problems	PDP	ADHD	Oppositional problems
CSHQ-total score		0.594**(0.000)**	0.53**(0.000)**	0.308**(0. 000)**	0.345**(0.000)**	0.361**(0.000)**
Affective problems	0.606**(0.000)**		0.652**(0.000)**	0.572**(0.000)**	0.525**(0.000)**	0.578**(0.000)**
Anxiety problems	0.525**(0.000)**	0.662**(0.000)**		0.558**(0.000)**	0.509**(0.000)**	0.578**(0.000)**
PDP	0.295**(0.000)**	0.571**(0.000)**	0.550**(0.000)**		0.466**(0.000)**	0.547**(0.000)**
ADHD	0.357**(0.000)**	0.533**(0.000)**	0.518**(0.000)**	0.480**(0.000)**		0.628**(0.000)**
Oppositional problems	0.366**(0.000)**	0.576**(0.000)**	0.580**(0.000)**	0.545**(0.000)**	0.623**(0.000)**	

Above the diagonal simple bivariate correlations, below the diagonal partial Pearson correlations, after controlling for age, gender and PIQ among the CSHQ total score and the CBCL DOS Scales. Significant correlations are highlighted in bold (value of *p* < 0.05).

Considering CBCL-DOS (Table 3), 64.9% (48/74) of the PS and 30% (27/89) of GS had a score over the borderline cut-off on one or more of the DOS, excluding the PDP scale given their clinical diagnosis of ASD. A logistic regression was performed to investigate the effects of the presence of sleep problems (GS or PS classification), age, gender and PIQ on the likelihood that children had at least one psychiatric comorbidity in addition to ASD. The logistic regression model was statistically significant, *χ*2(4) = 21.79, *p* < 0.0001. The model explained 16% (Nagelkerke *R*^2^) of the variance in the presence of comorbidity and correctly classified 67% of cases. As shown in Table 5, analysis reveals that being a PS increases the odds 4.3 times of having at least one psychiatric comorbidity in association with ASD, with a 95% CI of 2.20 to 8.39. No statistically significant association was found with the other variables included in the model.

**Table 5:** Logistic regression model of having one or more psychiatric comorbidities.

Variables	*B*	SE B	Wald *χ^2^*	*p*	OR	95% CI OR
Comorbidity						
Gender	−0.08	0.41	0.45	0.83	0.91	[0.41, 2.04]
Age	0.02	0.01	2.36	0.12	1.02	[0.99, 1.05]
PIQ	−0.00	0.00	0.02	0.88	0.99	[0.98, 1.01]
Group	1.45	0.34	18.27	**<0.0001**	4.29	[2.20, 8.39]

Significant comparisons are highlighted in bold (*p* < 0.05).

Finally, as shown in Table 6, we found differences between the sleep groups on the RBS-R subscales. The PS group showed higher scores on all subscale, except for the Self Injurious (value of *p* = 0.25), and on the Total Score when compared to the GS group (all value of *p*<0.05).

**Table 6:** Differences in RBS-R scales between poor and good sleepers.

	Good sleeper (*n* = 89)	Poor sleeper (*n* = 74)	*t*-test	Value of *p*	Effect size
Stereotypic behavior					
*M* (SD)	6.22 (4.34)	8.15 (5.97)	*t*_(161)_ = −2.37	***p* = 0.02**	*d* = 0.36
Self-injurious					
*M* (SD)	1.40 (2.31)	1.89 (3.08)	*t*_(161)_ = −1.15	*p = 0.25*	*–*
Compulsive					
*M* (SD)	2.02 (2.50)	3.19 (2.62)	*t*_(161)_ = −2.89	***p* = 0.004**	*d* = 0.45
Ritualistic					
*M* (SD)	4.36 (4.51)	6.22 (5.60)	*t*_(161)_ = −2.34	***p* = 0.02**	*d* = 0.36
Restricted					
*M* (SD)	2.01 (1.94)	2.77 (2.15)	*t*_(161)_ = −2.32	***p* = 0.02**	*d* = 0.36
Total score					
*M* (SD)	16.06 (12.21)	22.31 (16.10)	*t*_(161)_ = −2.81	***p* = 0.005**	*d* = 0.43

Significant comparisons are highlighted in bold (*p* < 0.05).

In order to assess the relationship between sleep and restricted and repetitive behaviors (RRBs) in the whole sample, we employed a simple bivariate Pearson correlation. We found a significant correlation between sleep problems and all subscales of the RBS-R (Table 7). This finding indicates that there is a strong association between sleep problems and RRBs in the sample studied, with all value of *p*s being less than 0.05. However, this association did not remain statistically significant after controlling for the degree of anxiety-related symptoms (Table 7), in line with previous research that showed anxiety could be a potential confounding variable (48) in the relationship between RRBs and sleep problems.

**Table 7:** Correlations between RBS-R subscale and CSHQ-total score, and partial correlations controlling for anxiety (DOS-CBCL anxiety problems).

RBS-R scores	Correlation		Partial correlation	
	*r*	*p*	*r*	*p*
Stereotypic behavior	0.26	**0.001**	0.09	0.21
Self-injurious	0.21	**0.006**	0.13	0.07
Compulsive	0.29	**0.000**	0.11	0.13
Ritualistic	0.29	**0.000**	0.07	0.35
Restricted	0.22	**0.004**	0.17	0.82
Total score	0.32	**0.000**	0.11	0.14

Significant correlations are highlighted in bold (value of *p* < 0.05).

## Discussion

The current study specifically investigated the presence and types of sleep problems in preschool children with ASD, as well as their relationship with clinical symptoms. We used the CSHQ parent questionnaire to assess the prevalence and severity of sleep problems through a quantitative measure. The use of parent-filled questionnaires such as the CSHQ has been shown to be a quick and easy-to-use tool for the early identification of sleep problems in both ASD and TD children (12, 13, 16, 25). According to previous investigations (11, 23), the established cut-off of 41 on the CSHQ could overestimate SD. Therefore, we decided to apply the more conservative cut-off of 48 to split our sample into two sub-groups based on the total score: good sleepers (GS) and poor sleepers (PS). As previously mentioned, the prevalence of sleep disturbance in ASD is extremely high, estimated from 15% up to 75% depending on the criteria used to identify sleep problems. However, only a few studies have focused on the preschool age group, identifying an even higher prevalence of SD in this population, ranging between 53 and 81% (10, 11, 49). In our sample, a significant proportion (46%) of children with ASD were found to have sleep problems as early as preschool age, even when applying a more stringent cut-off in the CSHQ. All CHSQ subscales were significantly higher in the PS group compared to the GS group. Our study confirms the high prevalence of sleep disturbance in the preschool population of children with ASD, which is significantly higher than that in preschool children with TD, as reported in previous studies (50–52). Focusing on this age group is important in terms of early identification because sleep problems tend to appear at a young age in ASD (53) and persist over time (54). Detecting SD in the ASD population before 6 years of age is fundamental for early intervention and to prevent behavioral issues related to sleep problems.

Our data also confirm recently reported findings in the literature indicating a strong association between SD and both internalizing problems (including anxiety, withdrawal, depression) and externalizing problems (including aggression, tantrums, and inattention) in ASD (21, 55). Specifically, looking at the number of psychiatric comorbidities, we found that more severe sleep problems increase the risk (four times) of having at least one psychiatric comorbidity in association with ASD, as measured through the CBCL. Sleep disruption may exacerbate associated psychiatric symptoms, or associated psychiatric comorbidities may worsen sleep problems already present in individuals with ASD (22). In the current study, the PS group had a higher score on CBCL across all domains with clinical relevance in internalizing, externalizing, and total problems in the syndromic scales. It is also noteworthy that unlike when we examine the ADOS scores, where the analysis did not find a difference in the severity of autism evaluated by clinicians between the two groups, parents reported on CBCL-PDP more problems related to the severity of autism in the PS group.

In particular, the correlational analysis we performed indicates that the total score on the CSHQ is associated with higher scores on anxiety problems, affective problems, pervasive developmental problems, attention deficit/hyperactivity problems, and oppositional deficiency problems on the DSM-Oriented subscales of CBCL, even when corrected for IQ, gender, and age.

These findings are in line with previous literature on this topic. For example, Sikora et al. reported in a sample of 1193 children with ASD aged 4–10 years that individuals with SD had significantly higher scores on the internalizing and externalizing behavior problems scales of the CBCL (23). Similar results were found by Park et al. in a study comparing 166 ASD children with their 111 unaffected siblings, demonstrating that children with ASD and sleep disturbances were more likely to exhibit aggressive behaviors, internalizing and externalizing behavior problems, and overall behavioral issues than peers without sleep issues (56). Focusing on younger ages, Roussis and colleagues recently reported that behavior problems and attention deficit were significantly greater in a group of ASD preschoolers with severe sleep problems than in peers with ASD but without sleep disorders (57).

Overall, evidence suggests that children with ASD and sleep difficulties have higher rates of Attention and Hyperactivity Disorder and Defiant Oppositional Disorder compared to children without sleep problems (7, 25, 57, 58). Generally, emotional or behavioral issues, including hyperactivity, aggression, anxiety, or mood disorders, may be the cause or consequence of sleep issues in children with ASD. An interesting finding that emerges from our data is that the correlation between SD and internalizing problems (specifically anxiety problems and affective problems) appears to be even stronger than that with externalizing disorders, in contrast to several studies in the literature (23, 51, 58–60). This finding is significant and worthy of further investigation. Future studies may attempt to characterize patients with externalizing and internalizing problems in more detail, trying to identify different subpopulations with specific clinical features.

Several studies suggest that anxiety and mood problems are also linked to SD in individuals with ASD (22, 47, 58, 61). Previous studies have found that children with higher levels of anxiety tend to exhibit increased repetitive and restrictive behaviors (RRBs) (62, 63), and have more difficulty sleeping (64). This suggests that anxiety may be a potential confounding variable in the relationship between sleep and RRBs in individuals with ASD (48). Although in our study, we found that the PS group displayed an increase in RRBs compared to the GS group, this difference disappears after controlling for the degree of anxiety-related symptoms, in line with previous research that showed anxiety could be a potential confounding variable (48). One possible explanation for this finding could be that RRBs might constitute a way to reduce perceived anxiety levels and to exert greater environmental control in patients with ASD (62, 63). According to this view, a higher number of RRBs would be an indicator of a higher level of anxiety, and higher levels of anxiety would correspond to greater difficulties in sleeping.

Furthermore, in our study, no significant association emerged between sleep disturbances and the extent of autistic symptoms measured through ADOS Score and level of cognitive development. Research has found a correlation between the severity of autistic symptoms and the presence of sleep problems in children with ASD (30, 65). It is not entirely clear what the relationship between sleep problems and autistic symptoms is in children with ASD. Some researchers have suggested that sleep problems may contribute to the severity of autistic symptoms, while others have proposed that the reverse may be true, with the severity of autistic symptoms contributing to the presence of sleep problems. In our study, we found no correlation between sleep problems and the severity of autistic symptoms, in agreement with other studies (61, 66).

Overall, while some evidence suggests a correlation between SD and the severity of autistic symptoms in children with ASD, more research is necessary to fully comprehend the connection between these two elements. Some studies have found that children with ASD and intellectual disability are more likely to experience SD than children with ASD alone (4, 5). However, we found no association between SD and developmental quotient, supporting the results of other studies (61, 65). These differences could be explained by the heterogeneity of the ASD populations analyzed by the different studies, particularly in terms of age groups and the tests used to assess cognitive development.

Sleep problems could be an early, although non-specific, symptom of ASD and are among parents’ first concerns (67), suggesting the presence of higher vulnerability during the early stages of life. Sleep disruption during developmental ages might be directly associated with abnormal brain development and could be an additional risk factor for cognitive and behavioral dysfunction (7). In this context, a recent study reported that sleep issues in the first year of life often occur before ASD diagnosis and are associated with atypical patterns of brain development in the hippocampal region (68). Several hypotheses have been formulated regarding the etiopathogenetic mechanisms of sleep alteration in ASD. According to some studies, the secretion of neurotransmitters, including serotonin, GABA, and melatonin, which are essential for establishing a regular sleep–wake cycle, may be altered (69, 70). Other studies suggest the possible involvement of genes implicated in the control of circadian rhythm (71, 72) or the presence of general disrupted sleep architecture in ASD (50, 70). Assuming that an intrinsic cause of disrupted sleep in ASD may be related to variations in brain wave organization and maturational development, further studies involving objective measurements of sleep, such as sleep EEG, polysomnography, or actigraphy, may be beneficial in investigating the underlying pathophysiological mechanisms (73).

Major limitations to our study are the use of predominantly subjective instruments such as parent-completed CSHQ, CBCL and RBS-R questionnaires. Such instruments may obviously be affected by parents’ personal perception of the issues. Future studies using objective parameters such as polysomnography-EEG and actigraphs will be needed. Another limitation is the possible presence of medical conditions underlying sleep disorders such as the presence of enlarged tonsils and / or adenoids causing obstructive sleep apnea: indeed, in the absence of specialized medical evaluations, we cannot exclude *a priori* the presence of underlying organic causes. Further medical investigations should be performed to rule out the presence of any underlying diseases. Moreover, a comparison of sleep problems and their impact on global functioning and emotional-behavioral problems between ASD and TD preschoolers, as well as between ASD and preschoolers with other neurodevelopmental disorders could be addressed in future studies.

To sum up, sleep problems are among the most frequently represented comorbid symptoms in children with ASD as early as preschool age. A wide number of literature reviews demonstrate that sleep disruption is link to emotional and behavioral issues in ASD, thus influencing children’s health, cognition, executive functions and consequently school performance besides having an important impact on the quality of life of families (3, 16, 21, 74, 75). Despite this, they are often not properly identified and treated in clinical practice (8, 21), as treatment guidelines to help parents manage challenging behaviors in children with ASD frequently fail to include sleep at all or only briefly address the topic. Identifying and providing correct treatment for sleep problems in ASD is crucial not only to improve sleep, but also for better daytime conducts and family functioning in this population. Practice guidelines and recommendations for the treatment of disrupted sleep behavior and insomnia in children and adolescents with ASD updated to 2017 have been provided by the American Academy of Neurology (76), but continuous upgrading of expert consensus statements is needed to deal with this disabling symptom.

## Data availability statement

The raw data supporting the conclusions of this article will be made available by the authors, without undue reservation.

## Ethics statement

The studies involving human participants were reviewed and approved by IRCCS Stella Maris committee. Written informed consent to participate in this study was provided by the participants’ legal guardian/next of kin.

## Author contributions

GD, MT, SC, and FM were involved in designing the study and drafting the initial manuscript. MT performed the data analysis. GD, MT, SC, GT, GP, FA, RI, and AC assessed the patients and collected the data. GD, MT, SC, GT, GP, FA, RI, AC, FM, FC, and RT contributed to editing the manuscript, providing critical feedback, and performing a thorough review. All authors have reviewed and approved the current version of the manuscript and take full responsibility for its contents.

## Funding

MT received funding from the GenPercept grant agreement (no. 832813). This study was partially funded by grants from the IRCCS Fondazione Stella Maris, including the Ricerca Corrente and the 5 × 1000 voluntary contributions from the Italian Ministry of Health, as well as by AIMS-2-Trials.

## Conflict of interest

The authors declare that the research was conducted in the absence of any commercial or financial relationships that could be construed as a potential conflict of interest.

## Publisher’s note

All claims expressed in this article are solely those of the authors and do not necessarily represent those of their affiliated organizations, or those of the publisher, the editors and the reviewers. Any product that may be evaluated in this article, or claim that may be made by its manufacturer, is not guaranteed or endorsed by the publisher.
